# Improving the *ex vivo* expansion of human tumor-reactive CD8 + T cells by targeting toll-like receptors

**DOI:** 10.3389/fbioe.2022.1027619

**Published:** 2022-10-31

**Authors:** Chenli Qiu, Jing Wang, Lingyan Zhu, Xiaobo Cheng, Bili Xia, Yanling Jin, Ran Qin, LinXia Zhang, Huiliang Hu, Jia Yan, Chen Zhao, Xiaoyan Zhang, Jianqing Xu

**Affiliations:** ^1^ Shanghai Public Health Clinical Center, Fudan University, Shanghai, China; ^2^ Zhongshan Hospital & Institutes of Biomedical Sciences, Fudan University, Shanghai, China; ^3^ Clinical Research Center, Obstetrics & Gynecology Hospital of Fudan University, Shanghai, China

**Keywords:** toll-like receptors, agonists, adoptive cell therapy, human PD1+ CD8^+^ T cells, cell proliferation, tumor cell killing

## Abstract

Toll-like receptors (TLRs) are important pattern recognition receptor(s) known to mediate the sensing of invading pathogens and subsequent immune responses. In this study, we investigate whether TLRs could be explored for the preparation of human CD8^+^ T cell products used in adoptive cell therapy (ACT). Following characterization of TLRs expression on human CD8^+^ T cells, we screened TLR-specific agonists for their ability to act in concert with anti-CD3 to stimulate the proliferation of these cells and corroborated the observed co-stimulatory effect by transcriptional profiling analyses. Consequently, we developed an optimal formulation for human CD8^+^ T cell amplification by combining CD3/CD28 antibody, interleukin 7 (IL-7), interleukin 15 (IL-15), and three agonists respectively targeting TLR1/2, TLR2/6, and TLR5. This new formulation performed better in amplifying PD-1+CD8^+^ T cells, a potential repertoire of tumor-reactive CD8^+^ T cells, from tumor patients than the conventional formulation. Importantly, the expanded CD8^+^ T cells showed restored functionality and consequently a robust anti-tumor activity in an *in vitro* co-culturing system. Together, our study established the utility of TLR agonists in *ex vivo* expansion of tumor-targeting CD8^+^ T cells, thus providing a new avenue toward a more effective ACT.

## Introduction

Pattern recognition receptors (PRRs) stand in the frontline of host immune response against invading pathogens. Such leading roles rely on the ability of PRRs to recognize pathogen-associated molecular patterns (PAMPs), thereby initiating a cascade of cell signaling that culminate in the induction of interferons (IFNs) for eliciting antiviral innate immunity as well as proinflammatory cytokines and chemokines that attract the immune cells to the site of infection (Takeuchi and Akira 2010). Toll-like receptors (TLRs) are an important family of PRRs that are highly conserved during evolution, accounting for the recognition of a wide array of diverse PAMPs featured by bacteria or viruses. Depending on cellular localization, TLRs can be classified into two primary subfamilies, the cell surface TLRs and the intracellular TLRs. The former includes TLR1, TLR2, TLR4, TLR5, TLR6 and TLR10, whereas the latter is comprised of TLR3, TLR7, TLR8, TLR9, TLR11, TLR12, and TLR13 (Oosenbrug et al., 2017). The two subfamilies also divide in labor, with the cell surface TLRs mainly dedicated to the recognition of microbial membrane components such as lipids, lipoproteins, and proteins while intracellular TLRs being involved in sensing non-self-nucleic acids and might also contributing to the autoimmunity when aberrantly activated by self-nucleic acids under disease conditions.

TLRs are transmembrane proteins and can be structurally divided into three domains: an ectodomain featuring multiple leucine-rich repeats (LRR) for ligand interaction, a transmembrane domain, and a cytosolic signaling domain, usually referred as TIR domain (Pandey and Agrawal 2006). Upon ligand binding, TLRs undergo conformational changes that promote their homodimerization or heterodimerization. For instance, depending on the identity of the bound ligand, TLR2 can act by forming homodimer or heterodimer with TLR1/TLR6 (Matsumoto et al., 2012). Following dimerization, TLRs then recruit one or more TIR domain-containing adaptor proteins (TIRAPs). As of date, a number of TIRAPs are identified, including MyD88 (myeloid differentiation primary-response gene 88), MAL (MyD88 adapter-like), TRIF (TIR-domain containing adaptor protein inducing IFN-β), and TRAM (TRIF-related adaptor molecule) (Takeda and Akira 2005). MyD88 functions as the primary TIRAP shared by all TLRs except TLR3, initiating a signaling cascade comprising IRAK4/IRAK1 complex, TRAF6, TAK1, MAP kinase and IκB kinase (IKK), which culminate in the activation of transcriptional factor NF-κ B and AP1 to upregulate the transcription and thus the production of proinflammatory cytokines. On the other hand, owing to their utilization of TRIF as the TIRAP to engage IRF-3 transcription factor, TLR3 and TLR4 activation are capable of inducing type I interferons (IFNs) in addition to proinflammatory cytokines (Krishnan et al., 2007). Thus, TLR activation can lead to the production of diverse cytokines, which subsequently act in not only establishing an antiviral state but also shaping the local milieu to facilitate the adaptive cell recruitment for the ultimate clearance of infected cells.

Consistent with their important roles in innate immunity, TLRs are expressed predominantly in innate immune cells, particularly in dendritic cells (DCs) and macrophages. Their expression can also be detected in non-immune cells such as fibroblast cells and epithelial cells. However, accumulating evidence suggests that the immunity-regulating activity of TLRs might also impact T cells directly (Komai-Koma, M.et al., 2004). TLRs were documented to be expressed on human T cells (Hornung et al., 2002). Mousa Komai-Koma et al. first reported that naïve human T cells upregulate TLR2 expression upon stimulation with anti-T cell receptor antibody and IFN-alpha, and consequently responds to bacterial lipopeptide, the TLR2 ligand, for IFN-gamma production. Peripheral blood-derived CD4^+^CD45RO^+^ memory T cells constitutively expressed TLR2, thus allowing a response to bacterial lipopeptide that results in robust IFN-gamma induction and marked enhancement of proliferation in cells cultured under the influence of IL-2 or IL-15. These findings point to the important contribution of TLR2 as a costimulatory receptor to both the antigen-specific CD4^+^ T cell development and the CD4^+^ T cell memory maintenance (Komai-Koma et al., 2004). Interestingly, mouse CD4^+^ T cells exhibited an activation-dependent induction of TLR-3 and TLR-9. Accordingly, treatment of poly(I:C) and CpG oligodeoxynucleotides (CpG DNA), respective synthetic ligands for TLR-3 and TLR-9, enhanced the survival of activated mouse CD4^+^ T cells without augmenting proliferation, whereas little effect was observed upon treatment with peptidoglycan and LPS, respective ligands for TLR-2 and TLR-4. This result, while revealing a species-dependent variation in CD4^+^ T cell regulatory activity of TLRs, strengthens the notion that the biological functions of different TLRs are at least partially imparted by dynamic gene expression pattern (Gelman et al., 2004).

Given the importance of CD8^+^ T cells in host immunity against viruses and tumors, the regulatory role of TLRs in these cells has also been studied. Most of these studies were carried out with murine CD8^+^ T cells. For instance, TLR2 triggering was found to enhance the proliferation, survival, and effector functions of murine CD8^+^ T cells, accompanied by a lowered threshold required for optimal cell activation (Cottalorda et al., 2006; Mercier et al., 2009). Consequently, functional memory cells are allowed to develop even upon partial activation by suboptimal TCR signaling. A synergy between TLR2 and TCR signaling in stabilizing IFN-γ mRNA was recently proposed as a mechanism underlying such threshold-lowering effect (Salerno et al., 2019). These findings were corroborated by the analyses of TLR2 knockout mice, which were characterized by a decreased frequency of memory CD8^+^ T cells, suggesting an antigen-independent mechanism for TLR2 to regulate CD8^+^ T cell memory formation. That is, TLR2 engagement of memory CD8^+^ T cells facilitates their proliferation and expansion in response to IL-7 stimulation (Cottalorda et al., 2009). Moreover, TLR2 signaling was shown to modulate the survival and fate of activated murine CD8^+^ T cells during viral challenge *via* the TLR2-MyD88 axis (Quigley et al., 2009). Our own studies further strengthened this notion, demonstrating that the intrinsic MyD88 pathway acts in TCR-independent manner to promote the acquisition of high functional avidity by VACV-boosted CD8^+^ T cells (Hu et al, 2014). Compared to TLR2, less is known about the roles of other TLRs in murine CD8^+^ T cells. In light of this knowledge gap, a more recent study demonstrated a synergistic effect between TLR7 agonist and anti-CD3 in *vitro* activation of murine CD8^+^ T cells, which occurs through the MyD88/Akt-mTOR pathway (Li et al., 2019).

There are only limited reports on TLR co-stimulation of human CD8^+^ T cells. A study using human neonatal CD8^+^ T cells indicated that co-administration of either Pam3SCK4 or flagellin, respective agonist for TLR2 and TLR5, led to augmented cellular response to TCR stimulation in terms of cell proliferation, memory formation, and cytokine production. Notably, a more robust enhancement was observed when the two agonists were applied together, compared to either agonist alone (McCarron and Reen 2009). On the other hand, another report proposed that TLR3 provides a co-stimulatory signal for the activation of human CD8^+^ effector T-cells (Tabiasco et al., 2006). Despite these studies, the information about the CD8^+^ T cell regulatory activity of specific TLRs remains scarce. In the context of such scarcity, the study presented here explored a practical use of TLR signaling for shaping the response of human blood-derived CD8^+^ T cells to TCR-mediated activation. This exploration led to the identification of a combination of TLR agonists that can effectively facilitate the *ex vivo* expansion of PD-1+ subpopulation of CD8^+^ T cells from blood of cancer patients, which represent a potential repertoire of tumor-reactive CD8^+^ T cells. Importantly, the expanded cells showed restored functionality and were able to kill cancer cells with high efficiency in an *in vitro* co-culture model. Our findings open new opportunities to improve the efficacy of ACT, and at the same time provide new clues for understanding the functions of TLRs in CD8^+^ T cell-mediated immunity.

## Materials and methods

### Human subjects

The study was reviewed and approved by the Ethics Committee of Shanghai public clinical center. All the participants, including healthy donors and cancer patients, signed informed consent form for their blood donations and the derived cell products to be used in this research project; all the participating cancer patients were enrollees of a clinical research trial project registered in ClinicalTrails.gov (Identifier: NCT03093688).

### TLR agonists and cytokines

Human TLR1-9 Agonist Kit, TLR1/TLR2 agonist Pam3CSK4, TLR5 agonist flagellin (FLA), and TLR2/TLR6 agonist FSL-1 were purchased from InvivoGen. Recombinant human IL-7 and recombinant human IL-15 were products of R&D Systems.

### Peripheral blood mononuclear cells (PBMCs) preparation, CD8^+^ T cells isolation and proliferation assay

For preparing PBMCs, 10 ml of whole blood was drawn from each donor and separated by Ficoll gradient centrifugation. The PBMC layer was collected and washed twice with PBS. The isolation of CD8^+^ T cells from PBMCs was performed by negative selection using EasySep Human T-Cell Isolation Kit (StemCell Technologies, Canada). The examination of the *ex vivo* proliferation of isolated CD8^+^ T cells was based on labeling cells with eFluor670 proliferation tracer (eBioscience). The labelled cells were then stimulated with Dynabeads (Invitrogen) coated with anti-CD3 (BD Biosciences) in the presence and absence of different TLR agonists, or Dynabeads coated with anti-CD3 plus anti-CD28 (BD Biosciences) as a positive control. On day 7, the cells were analyzed by flow cytometry (BD LSRFortessa) and the percentage of proliferated cells was determined according to the eFluor670 dilution. To determine the dosage effect of TLR agonists, co-stimulation was carried out under three concentrations of 1 ng/ml, 10 ng/ml, and 100 ng/ml.

### PD-1+ CD8^+^ T cells isolation and *ex vivo* expansion

For isolation of blood-derived PD-1+ CD8^+^ from healthy donors and cancer patients, PBMCs were first isolated and, after staining with CD3-BV421 (BD Pharmingen), CD8-APC-H7 (BD Pharmingen), PD-1-PE-CF594 (BD Pharmingen), sorted into 96-U-well cell plate at 5,000–10,000 cells per well using a FACS Aria II cell sorter (BD Biosciences). The cells were allowed to settle for 1 hour before the medium was replaced with fresh x-vivo 15 medium, to which anti-CD3/anti-CD28 beads (cells:beads = 1:1) were added together with recombinant human IL-7 (R&D, 20 ng/ml), recombinant human IL-15 (R&D, 20 ng/ml), Pam3CSK4 (InvivoGen, 100 ng/ml), FSL-1(InvivoGen, 10 ng/ml), and flagellin (InvivoGen, 10 ng/ml). After 7 days’ incubation, the cells were transferred to a 24-well plate for further expansion under the same culturing/stimulation conditions. The cell cultures were subsequently expanded to T25 flask and then T75 flask at appropriate time points depending on the cell growth rate, with the final cell products normally harvested between 21 and 28 days after the initial seeding.

### Immunophenotyping, intracellular cytokine staining, and cytokine release assessment

For surface staining, cells were stained in FACS buffer (PBS supplemented with 2% bovine serum albumin) sequentially with a fixable viability dye and a combination of the following antibodies: CD3-ECD (IM2705U, Beckman coulter), CD8-BV711(563677, BD Pharmingen), CD4-percp-cy5.5 (300530, Biolegend), CD14-BV786(563699, BD Pharmingen), TLR1-FITC(sc-47709FITC, Santa Cruz), TLR2-PE-Vio770 (130-099-021, Miltenyi), TLR4-BV421 (312811, Biolegend), TLR5-Alexa Fluor 700 (NBP2-24787AF700, NOVUS biology), TLR6-DyLight 350 (NB100-56536UV, NOVUS biology), CD3-BV421(562426, BD Pharmingen), CD8-APC-H7(580179, BD Pharmingen), PD-1-PE-CF594(565024, BD Pharmingen), CTLA-4-APC(555855, BD Pharmingen), ICOS-FITC(313505, Biolegend), 4-1BB-PE-Cy7 (309818, Biolegend).

The measurements of the effector responses of the expanded CD8^+^ T cells to anti-CD3/anti-CD28 stimulation was carried out in 96-well plates. CD107a-Percp-Cy5 (328616, Biolegend) was added to each well and the cells were then stimulated with anti-CD3/anti-CD28 for 1 hour, followed by treatment with 10 μg/ml of secretion inhibitor brefeldin A (Sigma) for 5 hours. The cells were subsequently fixed and permeabilized using a Cytofix/Cytoperm kit (BD Biosciences) according to the manufacturer’s instructions. Following this, the cells were stained for 30 min with IFN-gamma-PE/cy7 (505826, Biolegend), Granzyme B-AlexaFluor647(560212, BD Pharmingen), and IL-2-APC-Cy7 (500342,Biolegend) before acquired on a LSR Fortessa flow cytometer (BD Biosciences) and analyzed using FlowJo software.

For cytokine release assessment, culture media of expanded cells were collected and assayed by Human Th1/Th2/Th17 CBA Kit (BD™ Cytometric Bead Array (CBA)) following manufacturer’s protocol.

### Real-time cell analysis

The antitumor activity of effector cells was evaluated in E-plate 16 by RTCA using the xCELLigence RTCA DP system (RTCA, ACEA Biosciences, United States). In brief, before seeding the target cells, each well was equilibrated with 100 μl of cell culture medium, followed by placing the plate into the RTCA DP station to obtain background cell-index (CI) values. After removal of the plate from the station, 100 μl of medium containing 1×10^4^ of A549 cells were added to each well. The plate was left undisturbed at room temperature for 30 min to allow even settlement of seeded cells and then returned into the RTCA DP station to start automatic real-time recording of CI. Twenty hours later, the plate was taken out of the station and 100 μl of medium was removed from each well before adding the effector cells ((1×10^5^ of PBMC or the expanded PD1+ CD8^+^ T cells) in a 100 μl volume at a 10:1 effector/target ratio. After equilibration at room temperature for 30 min, the plate was placed back into the station and data acquisition resumed.

### RNA-seq analysis

CD8+T cells isolated from PBMC were cultured under different stimulatory conditions for 24 h and then collected in RNAzol (MolecularResearch Center). The resulting samples were stored at −80°C until processed to purification of total RNA using Direct-zol RNA miniprep kit (Zymo Research). The quantity and quality of RNA samples were determined using Agilent RNA chips on an Agilent 2,100 bioanalyzer (Agilent, United States). The RNA-seq library preparation and sequencing were conducted by Jinweizhi Biotechnology Co., Ltd. (Suzhou, China). Pair-wise comparisons of transcriptomic data were performed using BRB Array Tools software (http://linus.nci.nih.gov/BRB-ArrayTools.html), with differentially expressed genes being identified according to the *p* value (*p* < 0.01) and fold change (fold change >2). DAVID Bioinformatics Resources was employed for gene ontology (GO) analysis.

### Statistical analysis

All the statistical analyses were performed using GraphPad Prism software (Prism V8.0, GraphPad). Two-tailed Student’s t-test was used to compare differences between two groups, one-way ANOVA followed by post hoc Tukey test was applied when comparing three or more groups. In all cases, a *p* value of less than 0.05 was considered statistically significant.

## Results

### TLRs are differentially expressed in human blood-derived CD8^+^ T cells

We first characterized the expression of surface TLRs in human CD8^+^ T cells derived from healthy individuals using flow cytometry with monocytes as control. As shown in Supplementary Figure S1, TLR2, TLR4 and TLR5 were readily detected in the majority of monocytes whereas only a small subpopulation of cells showed expression of TLR1 and TLR6. The frequencies of positive cells were 4.18 ± 2.07%, 96.52 ± 1.47%, 60.90 ± 22.36%, 99.85 ± 0.25%, and 1.42 ± 0.87%, respectively for TLR1, TLR2, TLR4, TLR5 and TLR6. This result is consistent with the notion that TLRs are highly expressed in innate immune cells and play an important regulatory role in innate immunity.

**FIGURE 1:**
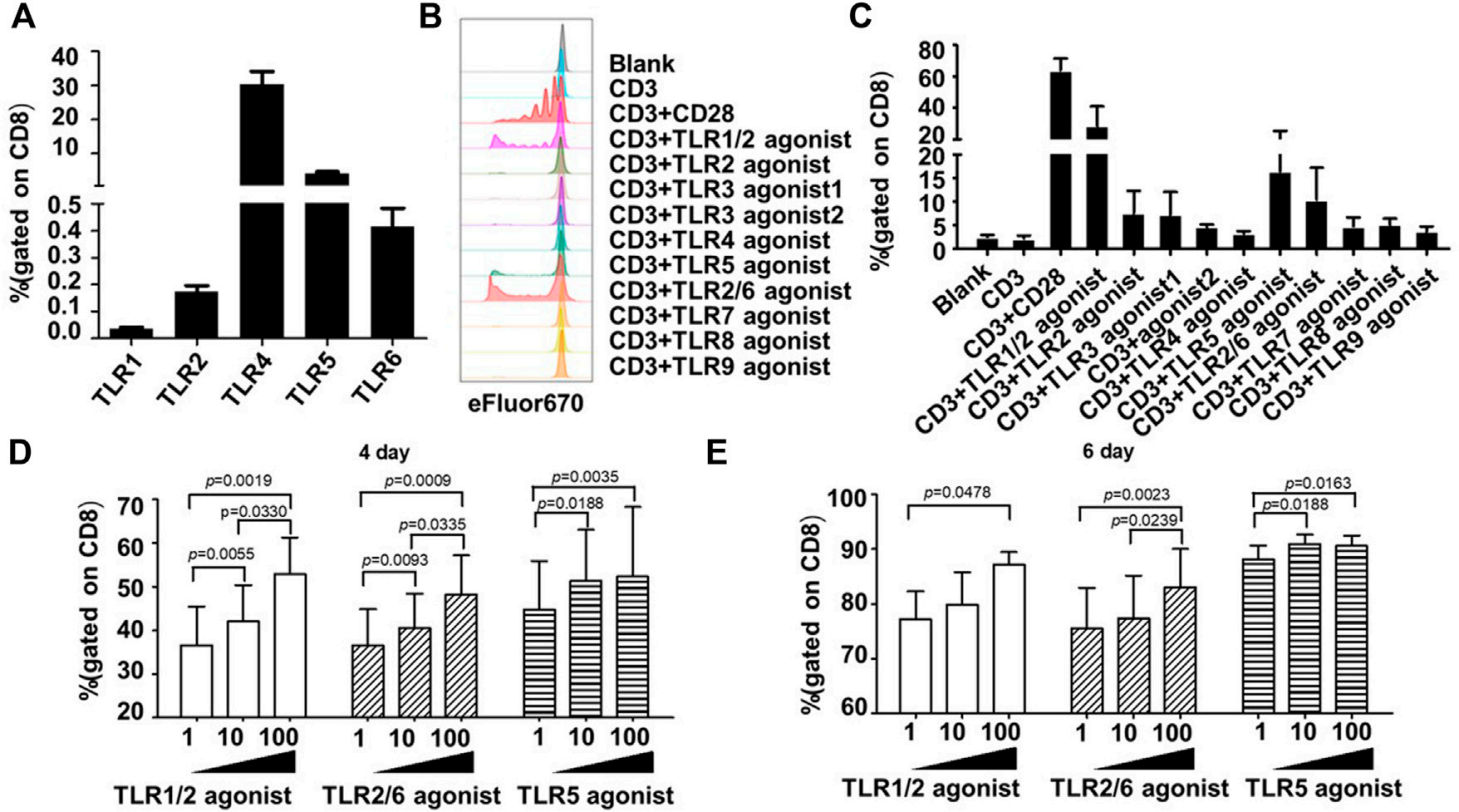
Co-stimulation with TLR1/2, TLR2/6, and TLR5 agonists enhances the proliferative response of human blood-derived CD8^+^ T cells to TCR triggering. **(A)** Expression profiles of surface TLRs in resting CD8^+^ T cells within human blood mononuclear cells (PBMCs). The T cell subpopulations of human PBMCs were identified by staining the cells with antibodies against CD3, CD4, and CD8; the TLR expression was measured by flow cytometry and expressed as the percentage of positively stained cells. Shown is aggregated data of 12 samples. **(B and C)** Co-stimulatory effects of different TLR agonists on the proliferation of human PBMC-derived CD8^+^ T cells. CD8+T cells were purified from human PBMCs, stained with eFluor 670, and then stimulated with ant-CD3 beads, anti-CD3/CD28 beads, or anti-CD3 beads coupled with the indicated TLRs agonists. Cells were harvested 7 days later and dilution of eFluor 670 was determined by flow cytometry to measure cellular proliferation, with representative flow cytometry plot and data summary (n = 3) shown in **(B)** and **(C)**, respectively. **(D,E)** Determination of optimal dosages of TLR agonists required for co-stimulation of *ex vivo* proliferation of human CD8^+^ T cells. CD8+T cells purified from human PBMCs were stimulated with anti-CD3 beads in presence of various concentration of TLR1/2 agonist, TLR2/6 agonist, or TLR5 agonist after labelling with eFluor 670. Flow cytometry analyses were performed on day 4 and 6 after stimulation to determine the cellular proliferation through assessment of eFluor 670 dilution. Shown is aggregated data of 3 samples.

In contrast, all surface TLRs were expressed at relatively lower levels in unstimulated human CD8^+^ T cells, with some of them such as TLR1, TLR2, and TLR6 being hardly detected. The frequency of positive cells for TLR1, TLR2, TLR4, TLR5 and TLR6 were 0.04 ± 0.02%, 0.17 ± 0.08%, 30.23 ± 13.68%, 4.09 ± 1.76%, 0.41 ± 0.24%, respectively (Figure 1A).

### TLRs triggering enhances the proliferation of human CD8^+^ T cells

Following the above analyses of TLRs expression, we examined the co-stimulatory activities of different TLR agonists on human CD8^+^ T cells. As TLRs may indirectly affect the CD8^+^ T cell activity *via* other immune cell subsets within PBMC, we purified human CD8^+^ T cells from PBMC samples from three healthy individuals to high homogenicity (>98%). These cells were then stained with cell proliferation dye eFluor 670, followed by continuous exposure to either anti-human CD3 antibody-coated magnetic beads in the absence or presence of specific TLR agonist, or magnetic beads coated with both anti-CD3 and anti-CD28 antibodies as a positive control for a successful T-cell activator. Cell proliferation was assessed by flow cytometry 7 days later. A total of eight TLR-specific agonists were analyzed, including Pam3CSK4 for TLR1/2, HKLM for TLR2, two TLR3 agonists in high molecular weight and low molecular weight Poly(I:C) (Poly(I:C) HMW and Poly(I:C) LMW respectively), LPS for TLR4, flagellin for TLR5, FSL-1 for TLR2/6, imiquimod for TLR7, ssRNA40 for TLR8, and ODN2006 for TLR9.

As shown in Figures 1B,C, among these agonists, the TLR1/2 agonist Pam3CSK4, the TLR5 agonist flagellin and the TLR2/6 agonist FSL-1 were able to cooperate with anti-CD3 in stimulating the proliferation of human CD8^+^ T cells, respectively resulting in an average of 16.93-, 9.29-, and 5.17-fold increase in the frequency of the proliferated cells relative to anti-CD3 alone. We further analyzed the dosage effects of the three agonists by administering the agonist in three concentrations (1 ng/ml, 10 ng/ml, 100 ng/ml). For TLR1/2 agonist and TLR2/6 agonist, the measurements of cell proliferation at day 4 and day 6 showed a dose-dependent response as the percentage of proliferated cells gradually increased with increasing the agonist concentration. Whereas for TLR5 agonist, albeit a dosage effect being observed on day 4, the three concentrations resulted in similar degree of stimulation of cell proliferation on day 6 (Figures 1D,E). Based on these results plus cost consideration, we chose 100 ng/ml, 10 ng/ml, and 10 ng/mL as the working concentration respectively for the TLR1/2 agonist, the TLR5 agonist, and the TLR2/6 agonist in the following study.

**FIGURE 2:**
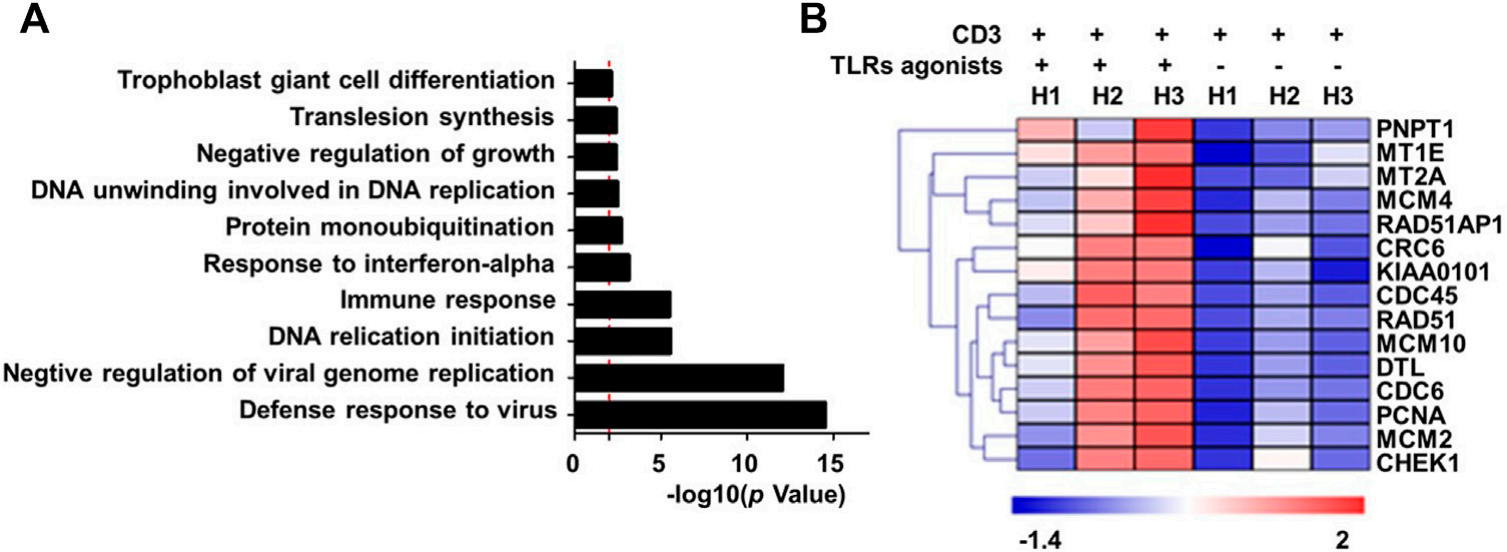
Co-stimulation with a combination of TLR1/2, TLR2/6, and TLR5 agonists enhances the transcriptional induction of proliferative-related genes in human blood-derived CD8^+^ T cells responding to TCR triggering. CD8+T cells purified from human PBMCs were stimulated with anti-CD3 beads in the presence or absence of a combination of TLR1/2 agonist, TLR2/6 agonist, and TLR5 agonist. Cells were harvested 24 h later for extracting total RNA, which was subjected to RNA sequencing. The differentially expressed genes showing >2-fold change between minus and plus TLR agonists groups were clustered based on Gene Ontology (GO) enrichment analysis **(A)**. Shown in **(B)** is the heatmap of representative proliferation-related genes that were identified to be co-stimulated by TLR agonists. H1 to H3 represent samples from three healthy individuals.

To explore the mechanistic aspect of the effect of TLRs co-stimulation on human CD8^+^ T cells, we prepared RNA from human CD8^+^ cells that were treated for 24 h with anti-CD3 alone or in combination with the three TLR agonists, for transcriptomic analyses. The Gene ontology (GO) enrichment analyses revealed that the genes differentially induced by co-stimulation with TLR agonists are mainly enriched in two functional categories: 1) virus-responsive genes alongside antiviral genes; 2) genes functioning in DNA replication initiation (Figure 2A). The first category is well expected from the action of TLR signaling as one of the most important pathways that mediate an antiviral response. The identification of the second category, on the other hand, corroborated our above finding that TLR co-stimulation can synergize with anti-CD3 in promoting the proliferation of human CD8^+^ T cells. Further analyses indicated that TLRs triggering upregulated a variety of genes with diverse roles in the process of DNA, consistent with an orchestrated regulation that supports robust proliferation (Figure 2B).

We further utilize RNA sequencing to profile the changes in RNA expression associated with combinatorial treatment of two TLRs relative to only anti-CD3 stimulation or treatment of three TLRs combined. The removal of any individual TLR agonist appeared to attenuate the stimulatory effect on gene expression, as indicated by a reduction in the total number of upregulated genes (Supplementary Table S1) and more importantly an overall impairment of the induction of representative proliferation-related genes (Supplementary Figure S2). Interestingly, the results also suggested that the contribution from different TLR agonists might vary, with the TLR2/6 absence seeming to have the least effect.

### A novel approach coupling TLR agonist to TCR stimulator allows robust *ex vivo* expansion of PD-1+CD8^+^ T cells from human peripheral blood

The efficacy of ACT depends on both the quantity and quality of infused cells. The more tumor-reactive CD8^+^ T cells in a cell preparation, the better chance it has in leading to an effective antitumor response after infusion into patients. Thus, it is optimal to enrich tumor-reactive CD8^+^ T cells, rather directly using the whole CD8^+^ T cell population, prior to *ex vivo* expansion. As such, tumor-infiltrating CD8^+^ T cells have been recently explored as a starting pool for producing therapeutic T cells because of its enrichment in tumor reactivity; however, this effort has been facing the challenge that a significant portion, if not the majority, of tumor-infiltrating CD8^+^ T cells undergo exhaustion *via* the expression of inhibitory receptors such as PD-1, due to the education by an inflammatory tumor microenvironment. An exciting finding along the same line was the identification of PD-1 as a reliable marker for the clonally expanded, tumor-reactive CD8^+^ T cell subpopulation (Gros et al., 2014). Although this finding was originally made with tumor-infiltrating CD8^+^ T cells, PD-1+ CD8^+^ T cells from peripheral blood, which can be more readily obtained than their counterpart within tumor, also likely represents a subpopulation rich in antitumor activity, from which more active ACT products could be generated than starting with a non-selected CD8^+^ T cell pool.

**FIGURE 3:**
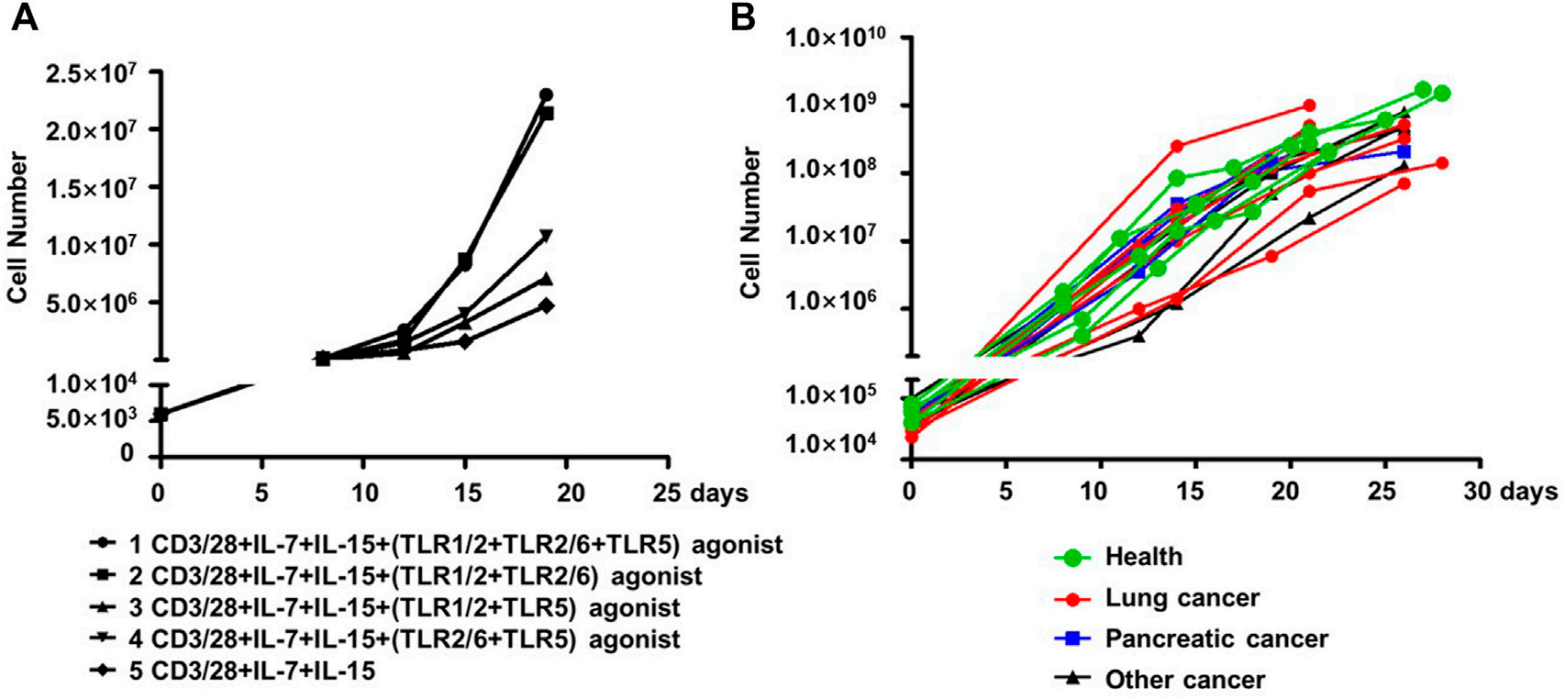
TLR agonists is an advantageous supplement benefiting the *ex vivo* expansion of human blood-derived PD1+ CD8^+^ T cells. **(A)** Exploration of optimal combination of TLR agonists in facilitating the *ex vivo* expansion of human blood-derived PD1+ CD8^+^ T cells. PD1+ CD8^+^ T cells isolated from blood samples of health individuals were continuously cultured for 21 days in medium containing anti-CD3/anti-CD28 beads and IL-7/IL-15, either alone or in the presence of different combination of TLR1/2, TLR2/6, and TLR5 agonists. Cell counting was performed at various time points throughout the culture period to generate the cell expansion curve. **(B)** Expansion curves of PD1+ CD8^+^ T cells derived from blood samples of cancer patients with different tumor types. Among the total of fifteen samples, seven were with lung cancer, four with pancreatic cancer, and the rest four with other types of cancers. Five samples from healthy individuals were also included as controls.

We assessed whether co-stimulation with different combinations of the three aforementioned TLR agonists could improve the expansion of human blood-derived PD1+ CD8^+^ T cells in an *ex vivo* culture system where cells are conditioned with IL-7 and IL-15 cytokines, in addition to CD3/28 coated magnetic beads. The employment of IL-7-IL-15 combination was based on previous studies demonstrating that IL-7/IL-15 effectively support the *ex vivo* expansion of naïve CD8^+^ T cells or CAR-T cells by promoting cell proliferation while suppressing apoptosis (Wallace et al., 2006). More importantly, IL-7-IL-15 showed a superiority over the traditionally used IL-2 in terms of their ability to expand CD8^+^ T cell subsets in a selective manner that better preserves the naivety and stemness of the resultant ACT products, consequently leading to a better expansion and engraftment after infusion (Xu et al., 2014; Zhou et al., 2019). We started our assessments on PD1+ CD8^+^ T cells derived from blood samples of healthy individuals. As shown in Figure 3A, the four combinations of the three TLR agonists varied in their ability to co-stimulate the cell expansion. In the presence of all three agonists, cells expanded by ∼4,800 fold in 20 days, which was 4.89-fold greater than that observed under the same culture condition without TLR agonists. Among the three combinations of two agonists, the TLR1/2/TLR2/6 agonist combination performed best, expanding cells to the similar extent as achieved by the combination of all three agonists, followed by the TLR1/2/TLR5 agonist combination and then the TLR2/6/TLR5 agonist combination, which only showed a moderate expansion-stimulating activity. Thus, we identified a new multi-ingredient recipe for the optimal *ex vivo* expansion of human blood-derived PD1+ CD8^+^ T cells, comprising anti-CD3/anti-28 antibody, IL-7/IL-15, and the combination of three TLR agonists respectively targeting TLR1/2, TLR2/6 agonists and TLR5.

We subsequently examined whether this recipe can be generally used to expand the blood-derived PD1+ CD8^+^ T cells from cancer patients. This examination involved fifteen blood samples from patients with lung cancer, pancreatic cancer, or other types of cancers, with a total number of 30,000-100,000 PD-1+CD8^+^ T cells being sorted from 1×10^7^ of PBMC followed by continuous culturing in a medium supplemented with anti-CD3/anti-28, IL-7/IL-15, and the three TLR agonists. Also included were five samples from healthy controls. After 21–28 days of culturing, almost all the cultures reached a size of 10^8^ cells or above, corresponding to a multiplication factor ranging 1,167 to 27027 with an average value of 6,400 (Figure 3B). Together, these results support TLR triggering as a general approach to improve the *ex vivo* expansion of human blood-derived PD1+ CD8^+^ T cells when acting together with anti-CD3/CD-28 and IL-7/Il-15.

### TLR-aided expansion of PD-1+CD8^+^ T cells resulted in functional cell products endowed with strong antitumor activity

**FIGURE 4:**
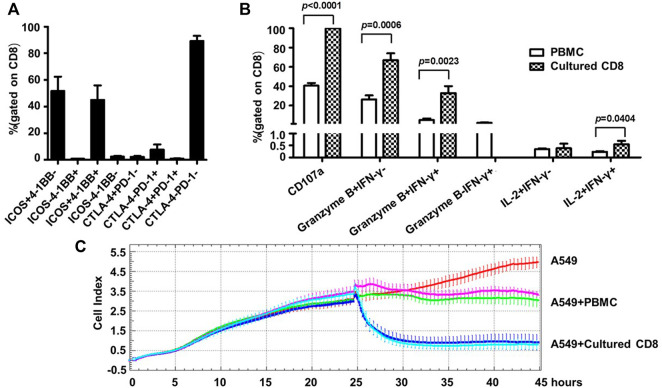
Human blood-derived PD1+ CD8^+^ T cells expanded by the new TLR agonists-aided method demonstrated strong effector and anti-tumor capacities. **(A)** Expression of co-stimulatory molecules (ICOS and 4-BB) and co-inhibitory molecules (CTLA-4 and PD-1) on the expanded PD1+ CD8^+^ T cells, assessed by flow cytometry. Shown is aggregated data of five samples. **(B)** Assessments of functional capacity of expanded PD1+ CD8^+^ T cells as effector cells. The expanded PD1+ CD8^+^ T cells were stimulated with anti-CD3/anti-CD28 beads in the presence of anti-CD107a, followed by treatment with secretion inhibitor brefeldin A for 5 h, and subsequently harvested for intracellular staining with markers granzyme B, IFN-γ and IL-2 before analyzing by flow cytometry (n = 5). **(C)** Tumor cell killing activity of expanded PD1+ CD8^+^ T cells. A549 cells were seeded at 1×10^4^ cells per well and incubated for 24 h before adding 1×10^5^ PBMC or the expanded CD8^+^ T cells. The co-culture was monitored for A549 cell index by RTCA system for 45 h. Shown are results of two representative samples.

Given the identity of PD-1 as a marker of exhaustion, we characterized the phenotypes as well as the activities of the above expanded PD-1+ CD8^+^ cells. For phenotype analyses, we assessed the expression of two important co-stimulatory molecules, namely ICOS and 4-1BB, and two important co-inhibitory receptors, namely CTLA-4, PD-1, by flow cytometry. Most of the expanded cells appeared to be positive for ICOS expression with the frequencies of ICOS+4-1BB- subset and ICOS+4-1BB + subset being 51.69 ± 23.99% and 45.05 ± 24.25% respectively, whereas cells expressing 4-1BB but not ICOS or lacking expression of ICOS and 4-1BB were scarce. In contrast, CLTA-4 or PD-1 expression was not detected in the majority of the expanded cells as cells positive for either CTLA-4 or PD-1 only accounted for approximately 11% of the whole population (Figure 4A). This contrasting expression pattern of co-stimulatory molecules *versus* co-inhibitory receptors suggested that the PD-1+CD8^+^ T cells were revigorated after expansion.

Next, we assessed the effector functions of the expanded cells. To this end, the expanded PD1+ CD8^+^ T cells were stimulated by anti-CD3/anti-CD28 and subsequently assayed for the expression of CD107a (a marker of degranulation), IFN-γ, granzyme B and IL-2 by flow cytometry; the corresponding parental PD1+ CD8^+^ T cells from cancer patient PBMC were included as the control. As shown in Figure 4B, the expanded cells showed superior effector functions compared with the control parental cells, as evidenced by marked increases in the frequencies of CD107a-positive cells (CD107a+, 99.89 ± 0.11% vs. 40.51 ± 6.98%), granzyme B single positive cells (granzyme B + IFN-γ-, 67.04 ± 15.86% vs. 26.23 ± 10.54%), as well as granzyme B/IFN-γ double positive cells (granzyme B + IFNγ+, 32.95 ± 15.85% vs. 4.96 ± 4.16%). After expansion, the percentage of IL-2+ IFN-γ+ cells also increased significantly, though its level was still very low. Interestingly, IFN-γ single positive cells (granzyme B- IFN-γ+), which appeared as a minor population in the parental cells, were undetectable in the expanded cells. The assessment of cytokine release *via* CBA confirmed robust production of IFN-γ by the expanded cells whereas the levels of other assayed cytokines including IL-2, IL-4, IL-6, IL-10, TNF, and IL-17a were too low for detection except for IL-10 and TNF, which were marginally detectable (Supplementary Figure S3).

Finally, we evaluated the antitumor activity of the expanded cells using an *in vitro* co-culture model system where human lung cancer A549 cells were used as target cells. The assay was initiated by adding PBMC or expanded PD1+ CD8^+^ cells to A549 cells, which were seeded 24 h earlier, at a effector/target (ET) ratio of 10:1. In the absence of effector cells, A549 cells reached a growth plateau about 42 h post seeding, with an average cell index of 4.79 (Figure 4C). Co-culturing with PBMC or the expanded CD8+T cells clearly inhibited the growth of A549 cells, but to different extents: there was little change in the cell index after the addition of PBMC, indicating a stalled cell growth; whereas the addition of expanded CD8^+^ T cells resulted in rapid decline of cell index, reaching the nadir approximately 5 h later. The data of the two studied samples was overall consistent: for sample 1, the final cell indexes attained in the presence of PBMC and CD8^+^ T cells were about 3.11 and 0.93, respectively, corresponding to an increase of killing rates from 35% to 81%; for sample 2, the same cell index numbers were respectively changed to approximately 3.49 and 0.79, from which killing rates were calculated as 27% *versus* 84%. We also conducted experiments in which cell products expanded from the same source of blood-derived PD1+ CD8^+^ T cells in the presence or absence of TLR agonists were compared for their cell killing capability. A representative result was shown in Supplementary Figure S4: the cell products raised with or without TLR agonists exhibited similar cell killing capability, suggesting that TLR agonists are likely to be capable of facilitating the expansion of PD1+ CD8^+^ T cells without compromising the cell killing capacity of expanded cells. Collectively, these assessments demonstrated that the TLR-aided expansion of PD1+CD8^+^ T cells from blood of cancer patients was able to consistently generate cell products endowed with robust tumor-killing capacity.

## Discussion

In addition to their well-known role of pathogen-sensing receptors in innate immunity, a growing body of evidence supported TLRs modulating the T cell-mediated adaptive immunity. This modulation reflects a potential synergy between TLR and TCR signaling in T cell activation as well as the subsequent memory formation. Based on these observations, we explored in this study whether TLR agonists can be utilized to improve the amplification of human blood-derived CD8^+^ T cells intended to be used for ACT therapy of cancer patients. Focusing on CD8^+^ T cells purified from human blood, this exploration started with analyzing expression of the five surface TLRs, followed by the demonstration of the cooperation of TLR1/2, TLR2/6 or TLR5 agonist and anti-CD3 in promoting cellular proliferation. Such proliferation-facilitating activities were further corroborated by transcriptomic analyses revealing that co-stimulation by the combination of the three TLR agonists led to a greater induction of cell proliferation-related genes. Consequently, we examined a new cell expansion formula, in which the three TLR agonists were combined with anti-CD3/CD28 antibody, interleukin 7 (IL-7), and interleukin 15 (IL-15), to amplify human blood-derived PD-1+CD8^+^ T cells, which represent a potential repertoire of tumor-reactive CD8^+^ T cells in cancer patients. With the new formula, we were able to more effectively amplify PD-1+ CD8^+^ T cells isolated from blood of not only health individuals but also cancer patients with different tumor types, as compared to the same formula while omitting the TLR agonists. Importantly, the resulting expanded CD8^+^ T cells were characterized by high expression of co-stimulatory molecules alongside low expression of co-inhibitory receptors, indicating a restored functionality. The potency of these cells in tumor killing was subsequently confirmed using a co-culture system where they showed superior activity in eliminating co-cultured A549 cells. Collectively, our study established the utility of TLR agonists in *ex vivo* expansion of tumor-targeting CD8^+^ T cells, and proposed it as a new tool for the development of a more effective ACT.

We chose to use flow cytometry for detecting cell surface expression of TLR proteins, which is more informative than the measurements of mRNA levels with respect to the responsiveness to signal through different surface TLRs. Our analyses revealed that, within the human blood derived CD8^+^ T cell population, an average of approximately 30% of the cells expressed TLR4, a frequency markedly higher than those of the rest four surface TLRs, among which TLR5 could be detected in about 4% of the cells whereas the population size for TLR1-, TLR2-, and TLR-6 positive cells were only 0.04%, 0.17%, and 0.41%, respectively. This expression pattern was in sharp contrast to the agonist-mediated profiling of the functionality of different TLRs in working with anti-CD3 to stimulate the expansion of human CD8^+^ T cells, which clearly indicated the TLR1/2, TLR2/6, and TLR5, but not TLR2 and TLR4, can signal the co-stimulation activity. Such discrepancy might be due to the possible variation in the detection sensitivity among the employed TLR1-speicific antibodies, which could result in the underestimation of the frequency of the cells expressing certain TLRs. Alternatively, the surface levels of TLR1/2, TLR2/6, and TLR5 protein are deemed so low in human CD8^+^ T cells that they are missed by the detection of staining antibodies; however, their triggering is still adequate to provide a co-stimulatory signal that collaborates with the TCR signal to promote the cellular proliferation. Interestingly, despite its higher expression on cell surface than other surface TLRs, TLR4 showed little activity in co-stimulating the proliferation of human CD8^+^ T cells. A notable difference between TLR4-mediated signaling and TLR2-or TLR5-mediated signaling is that the former is bifurcated into activation of both MyD88-and TRIF-dependent pathways whereas the later majorly only engages MyD88-dependent pathway (Nouri et al., 2021). This mechanistic difference could also contribute to the difference in cellular readouts of different TLR agonists. It should be noted that our data do not exclude the possibility that TLR4 is not involved in the modulation of human CD8^+^ T cells as our measurement focused solely on the effect on cellular proliferation, it is still likely that TLR4 might regulate other aspects of human CD8^+^ T cell biology, which merits further investigation in future studies. We also found that agonist-mediated triggering of TLR3, an endosomal TLR, failed to co-stimulate the cellular proliferation of human CD8^+^ cells when combined with anti-CD3. Whether surface TLRs are more favorable targets than endosomal TLRs to be harnessed for expanding human CD8^+^ T cells *ex vivo* needs to be addressed in future experiments.

To gain insights into how TLR signaling shapes the response of human CD8^+^ T cells to TCR triggering, we interrogated the transcriptional profiling of human CD8^+^ T cells upon activation by TCR triggering in the presence or absence of the combination of the three identified co-stimulatory TLRs agonists. Among the genes differentially upregulated by the co-stimulation with TLR agonists, we identified an enrichment of genes involved in DNA replication, corroborating their effects in promoting cellular proliferation. With our primary focus being on the exploration of TLR for a practical use in the preparation of human CD8^+^ T cells for ACT, we did not seek to further determine the molecular mechanisms underlying the upregulation of proliferation-related genes by TLR signaling. TLR signaling is known to activate MAP kinase, a key regulator of cell proliferation, downstream of the MyD88-TRAF6 axis. Thus, it is tempting to speculate that the activation of MAP kinase might be a key molecular event responsible for the enhancing effect of TLR signaling on the transcription of proliferation-related genes.

Current *ex vivo* expansion system of human CD8^+^ T cells or CAR-T cells is constructed following the theory that maximal T cell activation requires three signals: 1) TCR triggering by antigen recognition; 2) co-stimulation mediated by costimulatory receptors and ligands; 3) cytokine-promoted differentiation and expansion. In the system, the first and second signals are respectively provided by anti-CD3/anti-CD28 bead stimulation whereas IL-2 takes the role of the third signal traditionally. Several recent studies have revealed the superiority of IL-7/IL-15 over IL-2 in *ex vivo* expanding human naïve or tumor-experienced CD8^+^ T cells as well as CAR-T cells. Such superiority is multi-faceted, exemplified by improved stemness maintenance potentially *via* mitigating telomere attrition that accompanies cell division. More importantly, CD8^+^ T cells expanded with IL-7/IL-15 demonstrated greater antitumor effects than those expanded with IL-2. Accordingly, we chose Il-7/IL-15 over IL-12 as the cytokine component utilized in our expansion system. An important strategy to improve the efficacy of ACT is starting with a pool of human CD8^+^ T cells enriched with anti-tumor activity, rather than the whole CD8^+^ population where tumor-reactive cells only occupy a small fraction. PD-1 has been widely accepted as a marker for both T cell activation and exhaustion, and it was demonstrated by Steven Rosernberg’s group with tumor and blood samples from cancer patients that PD-1+ CD8^+^ T cells represent patient-specific, tumor-reactive CD8^+^ T cell repertoire marked by an ability to recognize a variety of tumor antigens (Gros et al., 2016). Thus, for the development of our method, we targeted blood-derived PD-1+ CD8^+^ T cells, which would be more valuable for ACT than bulk CD8^+^ T cells while more readily obtained than tumor-infiltrating CD8^+^ T cells.

The optimization of our cell expansion protocol was conducted on PD-1+ CD8^+^ T cells isolated from blood samples of healthy individuals. By comparing the expansion enhancing effect of different combinations of the three identified TLR agonists, we found that the combination of all three TLR agonists consistently resulted in the greatest enhancement. One plausible explanation for this finding is that the starting PD-1+ CD8^+^ T cells, though with high purity, is still a heterologous population in terms of TLR expression, with a specific TLR being only expressed on a subset of cells. In this scenario, all three agonists together would be expected to deliver co-stimulatory signals to more cells for proliferation than one or two agonists in combination. In addition, there might be crosstalk between signaling pathways activated by the three agonists, as well as with those transduced through CD3 and CD28, thus resulting in an additive and even synergistic effect in the promotion of cell proliferation. The fact that TLR agonists can exert their expansion-enhancing effect alongside the CD28 co-stimulation supports the notion that simultaneous engagement of multiple co-stimulatory signals with distinct downstream pathways might be an effective approach to optimize T cell activation. Indeed, by generating molecular maps and logical models for both TCR and TLR5 signaling pathways to pinpoint the aspect of their potential crosstalk, a most recent study suggested that TLR5 and CD28 signaling function similarly in boosting the TCR signaling, although differing in the underlying mechanisms (Rodriguez-Jorge et al., 2019).

We subsequently demonstrated the effectiveness of the optimized protocol in *ex vivo* expansion of blood-derived PD-1+ CD8+T cells from cancer patients. All the cultures showed an effective expansion after 28 days’s culturing with an average expansion rate of 6400-fold. As the patient donors are diversified in terms of tumor types, this result strongly supported the generality of our method. Importantly, the phenotype and functional analyses revealed the strong anti-tumor potential of the expanded cells. These cells were characterized by strong expression of co-stimulatory molecules as the majority being classified as either ICOS+ 4-1BB- or ICOS+ 4-1BB + cells; in contrast, only a small portion of them had detectable PD-1 expression, in sharp contrast to the starting cell population which was selected for PD-1 expression. Given the correlation between PD-1 expression and the exhaustion of tumor-experienced CD8^+^ T cells, the expanded CD8^+^ T cell appeared to gain a status that renders them ready for activation. This notion was strengthened by two functional demonstrations of the expanded cells: 1) compared to their PBMC counterparts, they showed augmented effector functions in response to anti-CD3/anti-CD28 stimulation, as indicated by the expression of CD107 activation marker and the production of granzyme B and IFN-γ; 2) they were able to effectively eliminate tumor cells in a co-culture system. A comprehensive characterization of these cells prior to and after activation would be beneficial for a better understanding of their potential application in cancer treatment, including their neoantigen-reactivity and perforin-producing capacity, as well as their original compositions of memory subpopulations and post-activation changes. Nevertheless, our results substantiated the use of TLR agonists in improving the efficacy of *ex vivo* expansion of human CD8^+^ T cells for ACT.

We recognize a potential limitation in our study. That is, we have not yet examined the *in vivo* functionality of the human CD8^+^ T cells expanded by our new method. One of our intended future studies would be using animal models to assess the *in vivo* anti-tumor functionality of our expanded CD8^+^ T cell products, either alone or in combination with other therapies. It should be noted that the efficacy demonstrated in the experimental arena might not be translated to clinical efficacy in treating cancer patients. Thus, the ultimate testimony of our method would come from using the expanded PD1+ CD8^+^ T cells directly for ACT therapy, an endeavor that we would like to pursue in the future. It is also of interest to determine whether the same combination of TLR agonists is useful for improving the *ex vivo* expansion efficacy of other types of therapeutic human T cells, including but not limited to tumor infiltrating CD8^+^ T cells and CAR-T cells. In addition, further optimization of our expansion procedures, which normally take between 21 and 28 days, to shorten the time needed is also imperative insomuch as its future exploration in industrial setting is considered, in light of recent remarkable achievement of Iovance Biotherapeutics in effective expansion of TILs within 16 days. Notwithstanding these limitations, our study underscores the co-stimulatory role of specific TLRs in the regulation of CD8^+^ T cell activation/proliferation and, by consequently presenting an improved protocol for expanding human blood-derived CD8^+^ T cells *ex vivo* that centers on the exploitation of TLR agonists, opens new opportunity to ease the efforts to prepare therapeutic T-cell products suitable for ACT.

## Data Availability

Raw reads of the RNA-sequencing data presented in this study have been deposited to NCBI’s SRA database (http://www.ncbi.nlm.nih.gov/sra/) under accession ID SRR21996460, SRR21996461, SRR21996462, SRR21996463, SRR21996464, SRR21996465, SRR21996466, SRR21996467, SRR21996468, SRR21996469, SRR21996470, SRR21996471, SRR21996472, SRR21996473, and SRR21996474.

## Supplementary material

Supplementary Figure S1 Expression profiles of surface TLRs in monocytes *versus* CD8^+^ T cells within human blood mononuclear cells (PBMCs). Shown are representative flow cytometry plots of expression of different surface TLRs in monocytes and CD8^+^ T cells from the same PBMC sample. FMO control: fluorescence-minus-one control.

Supplementary Figure S2 Comparison of the three TLR agonists cocktail, different combinations of two TLR agonists, and anti-CD3 alone on stimulating the expression of representative proliferation-related genes in blood-derived CD8^+^ T cells. Related to Figure 2B.

Supplementary Figure S3 Measurements of cytokine release by expanded CD8^+^ T cells. The levels of indicated cytokines in the culture medium of expanded CD8^+^ T cells were assessed by ELISA. Related to Figure 4.

Supplementary Figure S4 TLR agonists exposure during *ex vivo* expansion had little effect on the cell killing capability of the expanded PD1+ CD8^+^ cells. Shown is the result of a representative experiment in which cell products expanded from the same source of blood-derived PD1+ CD8^+^ T cells in the presence or absence of TLR agonists were compared for their cell killing capability in the *in vitro* co-culture system, measured by real-time cell analysis (RTCA). Related to Figure 4C.

Supplementary Table S1 Numbers of differentially regulated genes in blood-derived CD8^+^ T cells responding to TCR stimulation in the absence and presence of different combinations of TLR agonists. Related to Figure 2.
