# Supplementary figures and images for: Improving the ex vivo expansion of human tumor-reactive CD8 + T cells by targeting toll-like receptors
Source: Front Bioeng Biotechnol. 2022 Oct 31;10:1027619. doi: 10.3389/fbioe.2022.1027619 (PMC9659750; doi:10.3389/fbioe.2022.1027619)

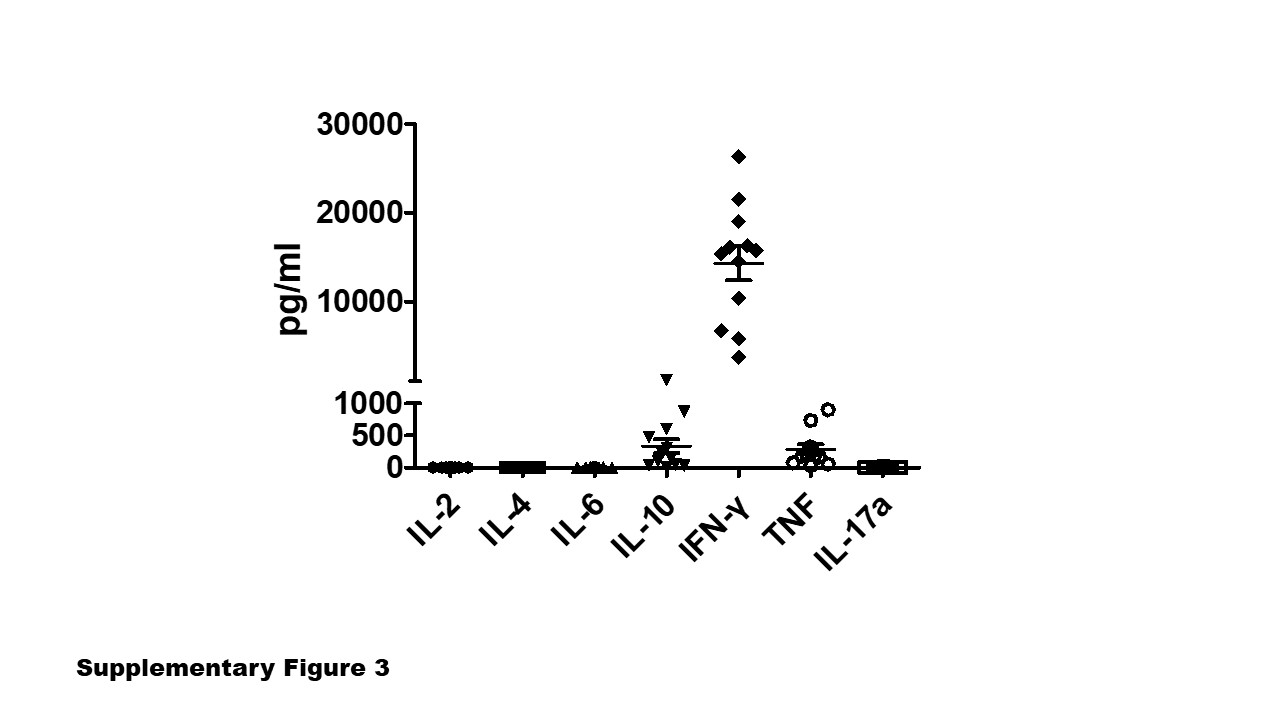

Supplement: Supplementary file 1 [file Image3.JPEG]

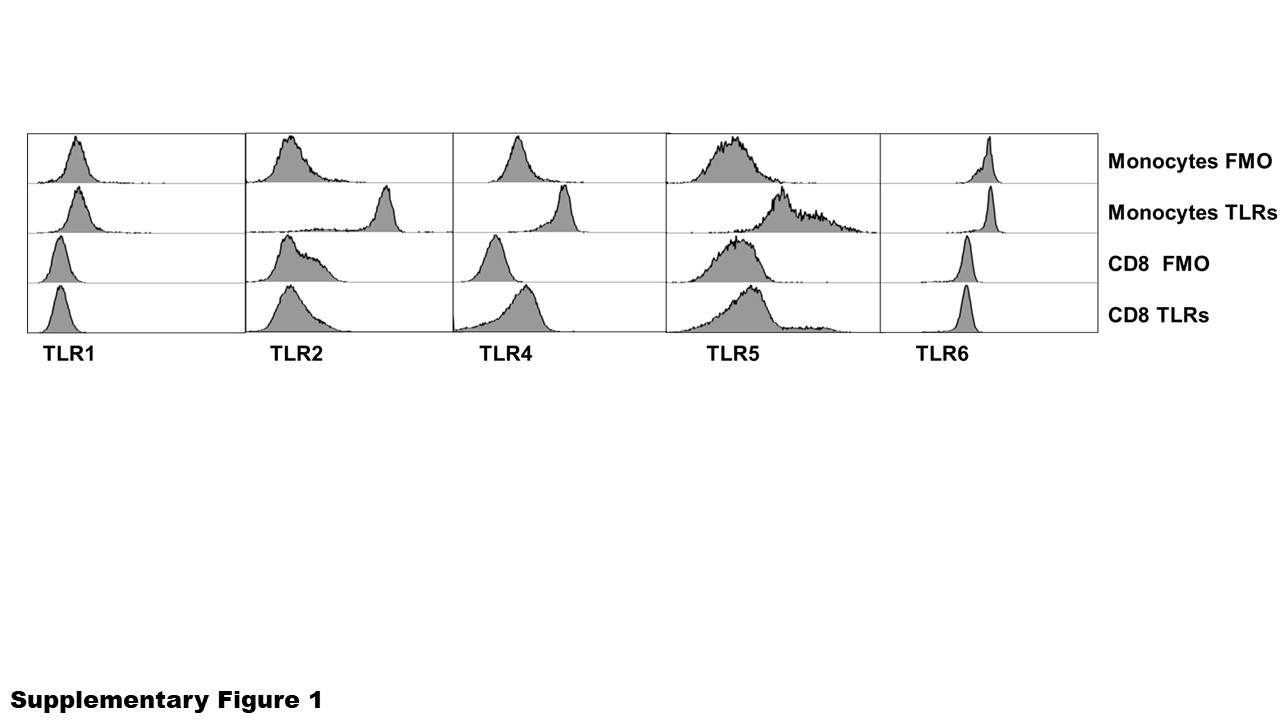

Supplement: Supplementary file 2 [file Image1.JPEG]

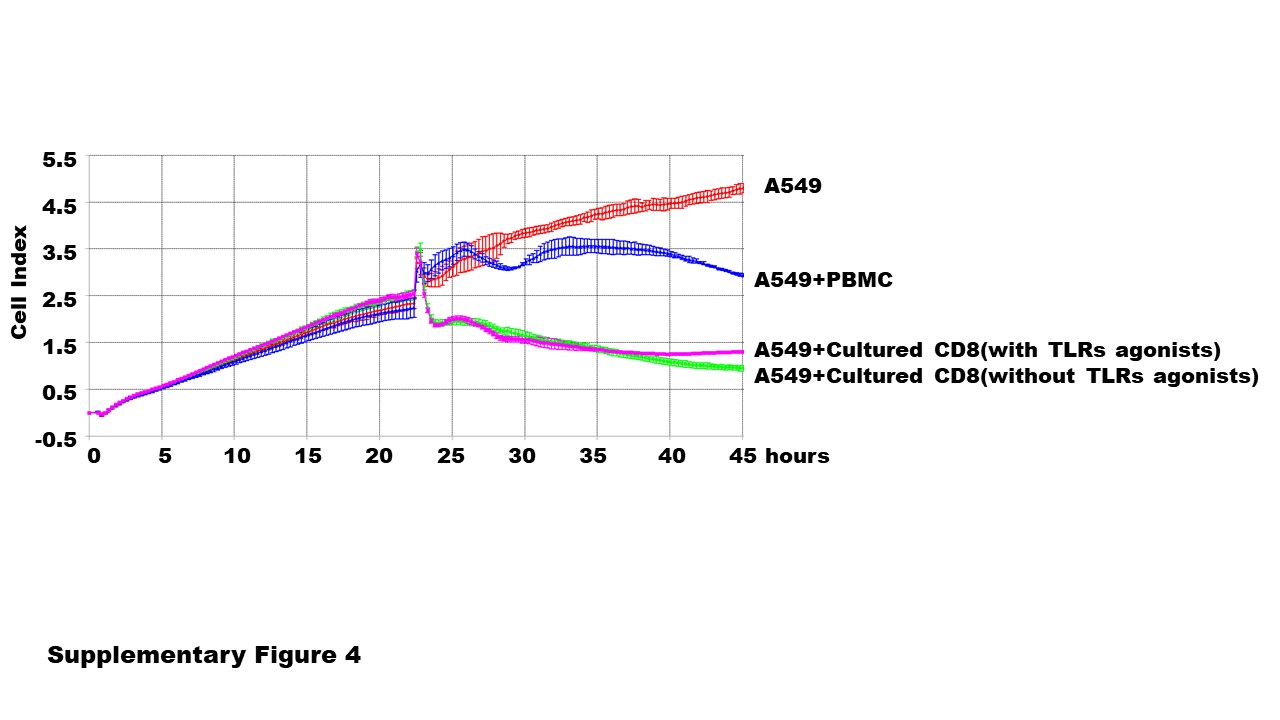

Supplement: Supplementary file 3 [file Image4.JPEG]

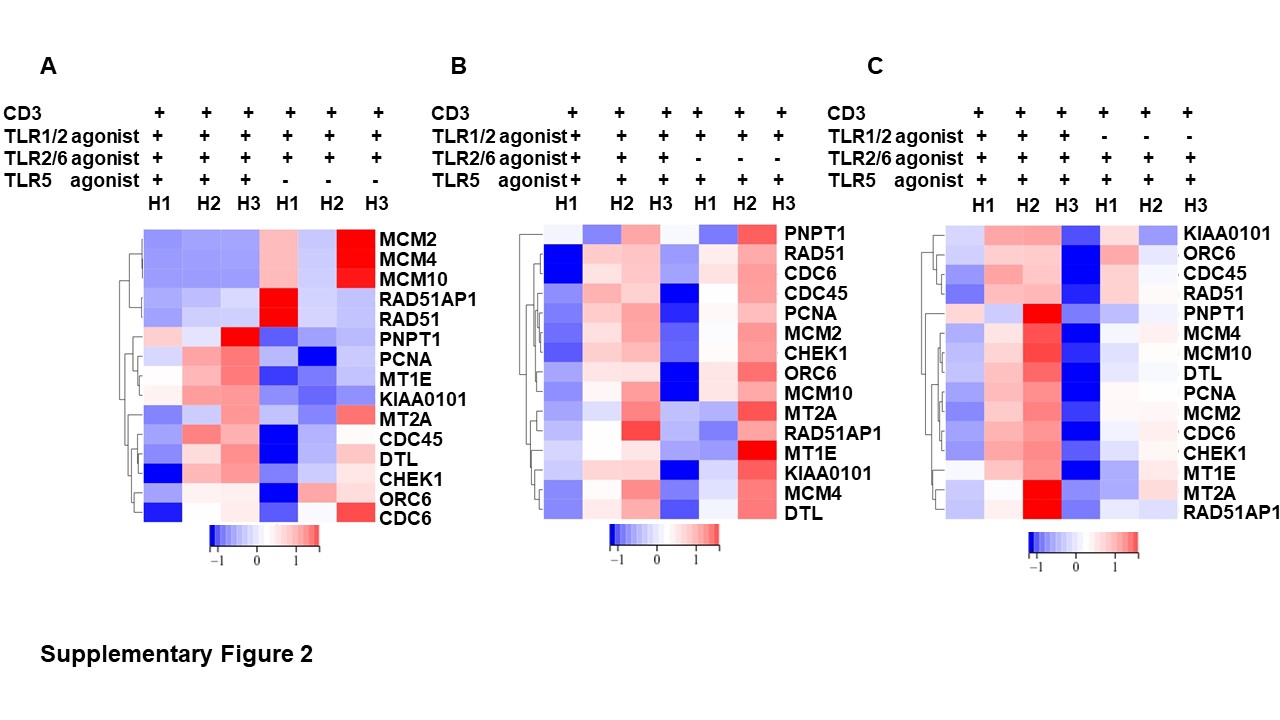

Supplement: Supplementary file 4 [file Image2.JPEG]

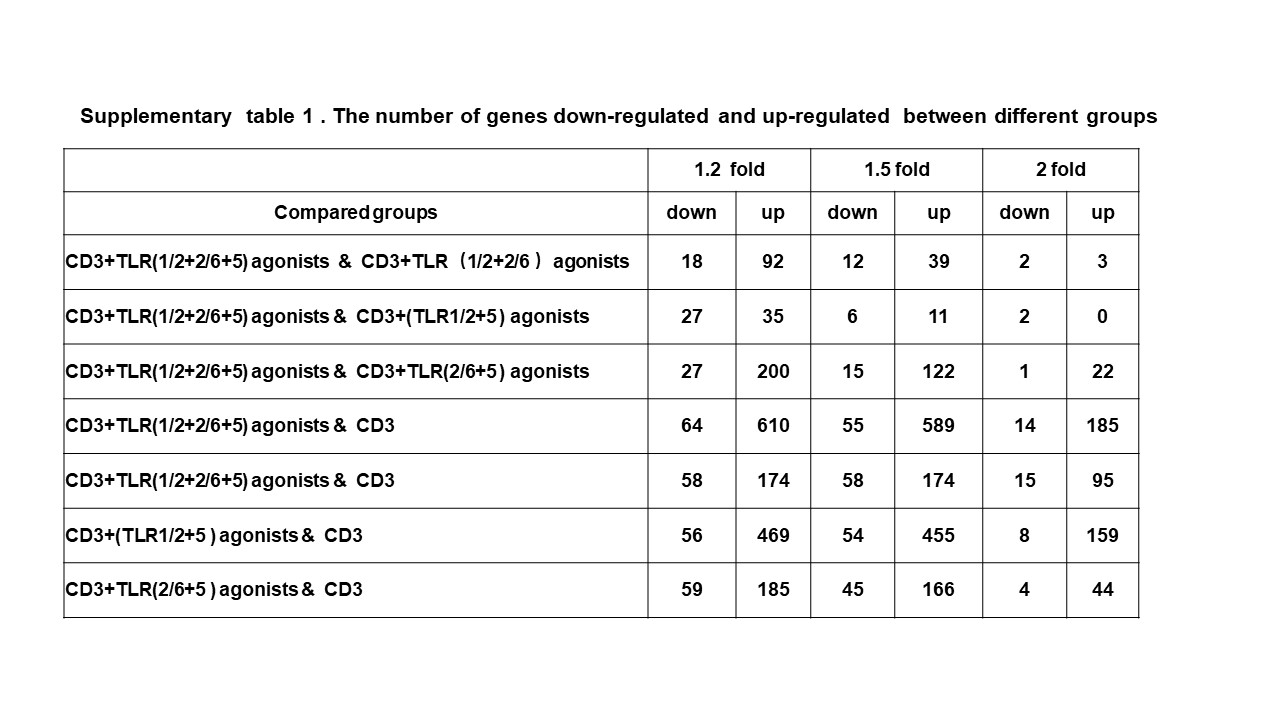

Supplement: Supplementary file 5 [file Image5.JPEG]
